# Iron-Deficiency Anemia Leading to Transient Ischemic Attacks due to Intraluminal Carotid Artery Thrombus

**DOI:** 10.1155/2013/813415

**Published:** 2013-09-12

**Authors:** H. Z. Batur Caglayan, B. Nazliel, C. Irkec, A. Dumlu, A. Filiz, M. Panpalli Ates

**Affiliations:** ^1^Department of Neurology, Faculty of Medicine, Gazi University, Besevler, 06500 Ankara, Turkey; ^2^Department of Neurology, Tokat State Hospital, 60100 Tokat, Turkey; ^3^Department of Neurology, Ankara Education and Research Hospital, 06340 Ankara, Turkey

## Abstract

Reactive thrombocytosis secondary to iron-deficiency anemia (IDA) is a rare but recognized cause of stroke. We report the case of a patient with iron-deficiency anemia presenting with multiple transient ischemic attacks (TIA) due to intraluminal thrombus of an internal carotid artery. The putative mechanisms underlying anemia and stroke syndromes are not completely understood, and it is believed that iron deficiency may cause ischemic stroke by several potential mechanisms. Thrombocytosis is often associated with iron deficiency, and microcytosis produces a reduction in the red cell deformability and could produce a hypercoagulable state. The platelet count and function observed in iron-deficiency anemia could act synergistically to promote thrombus formation, especially in the setting of an underlying atherosclerotic disease. The presence of floating thrombus in a patient with clinical and MRI evidence of stroke represents a significant therapeutic dilemma and requires immediate decision about treatment.

## 1. Introduction

Stroke can occur at any age, and although the majority occurs over the age of 65, nearly one-fourth of strokes occur in people under the age of 65. Some common causes in this population include cardioembolism, hematologic disorders, substance abuse, trauma, dissections, oral contraceptive use, connective tissue disorders, pregnancy, postpartum states, and migraine [1].

Reactive thrombocytosis secondary to iron-deficiency anemia (IDA) is a rare but recognized cause of stroke [2]. Here, the case of a patient with iron-deficiency anemia presenting with multiple transient ischemic attacks (TIA) due to intraluminal thrombus of an internal carotid artery is reported.

## 2. Case Report

**Figure 1:**
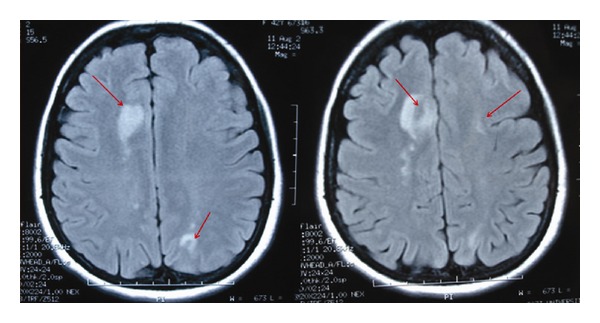
Brain MRI (FLAIR scans) with subacute ischemic lesions in left frontal and bilateral parietal regions.

A 41-year-old female patient was admitted to our hospital with transient numbness and weakness of the left and right upper and lower extremities that lasted less than 1 minute and had occurred twice on the day of admission. She also complained of having several of these attacks on the day prior to her admission to the hospital. Her past medical history was remarkable for anemia and menorrhagia, but she was not taking any medications, including iron replacement or oral contraceptives. The patient had no established risk factors associated with cardiovascular diseases except for smoking and her family history was negative for neurological or hematologic diseases. She was transferred to the neurology clinic for further evaluation. Her general physical, neurological, and fundoscopic examinations revealed nothing abnormal. Laboratory analyses revealed an iron-deficiency anemia with a hemoglobin level of 7.5 g/dL (13–17), a mean corpuscular volume (MCV) of 58 fL (80–92), a mean corpuscular hemoglobin (MCH) level of 16.75 pg (27–31), and a platelet count of 450 × 10 E3/*μ*L (130–400). Her serum iron level was 12 *μ*g/dL (25–156), total iron-binding capacity was 427 *μ*g/dL (135–526), ferritin level was 12 ng/mL (7–26), and saturation was 2.8%. The patient's lipid panel was normal: low-density lipoprotein (LDL) levels of 106 mg/dL (60–130) and high-density lipoprotein (HDL) levels of 64 mg/dL (30–80). Her other laboratory parameters, including white blood count, chemistry panel, urea, and creatine, were also within normal limits, and her electrocardiogram and chest X-ray were normal. Thrombophilia studies were negative following tests for antiphospholipid antibodies (anticardiolipin, IgG, and IgM antibodies), and no evidence of lupus anticoagulant was detected. Protein C, protein S, antithrombin III, fibrinogen, vitamin B12, folate, homocysteine levels, prothrombin, and partial prothrombin levels were within normal ranges, and the prothrombin gene variant G20210 and Factor V Leiden mutations were not detected. Antinuclear antibody, anti-DNA antibody, and antineutrophil cytoplasmic antibody tests were also negative. A transthoracic echocardiogram showed no evidence of septal defects or a cardiac source of emboli. The patient could not tolerate transesophageal echocardiography, so it was not performed. Brain magnetic resonance imaging (MRI) revealed a subacute left parieto-occipital, right frontal, and bilateral infarction (Figure 1).

**Figure 2:**
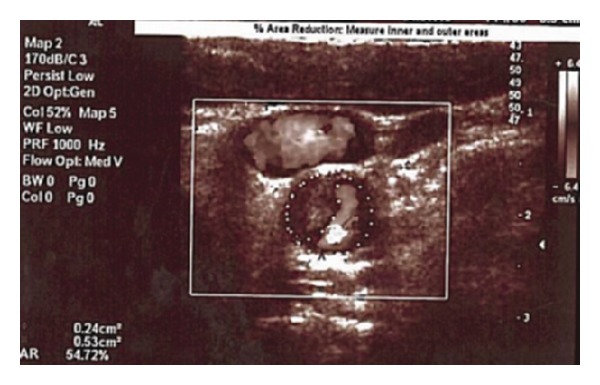
Carotid doppler ultrasonography shows left carotid artery with thrombus formation.

 A carotid artery doppler ultrasonography (USG) revealed the presence of an acute thrombus formation on the left internal carotid artery bifurcation (Figure 2).

No abnormality was present on the right internal carotid artery. A neck magnetic resonance angiography (MRA) revealed the presence of a signal void on the bifurcation of the left internal carotid artery and reported it as related to the thrombus formation detected on the carotid USG. Right carotid and bilateral vertebral arteries revealed no abnormalities following the neck MRA.

The iron deficiency was attributed to her menorrhagia. She received oral iron replacement and was anticoagulated with heparin, later converted to warfarin, with the aim of achieving an International Normalization Ratio (INR) of 2 to 3. A follow-up examination performed 1 month later demonstrated that the thrombus formation had resolved, and a carotid doppler USG revealed normal findings in the left internal carotid artery. 

## 3. Discussion 

Carotid artery disease is a common cause of cerebral dysfunction. Pathophysiologically carotid stenosis, occlusion, ulceration, dissection, inflammation, mural thrombus, and trauma have all been implicated. The imminent danger of a carotid clot is either distal embolization or progression to occlusion. The neurologic symptoms and signs may be related to either regional flow insufficiency or embolisation or both [3].

Iron-deficiency anemia (IDA), defined as a decreased total body iron, is characterized by microcytic hypochromic erythropoiesis with low serum iron and ferritin with an elevated total iron binding capacity [4].

Iron-deficiency anemia has been associated with papilloedema, idiopathic intracranial hypertension, venous sinus thrombosis, and ischemic stroke [5–9]. The putative mechanisms underlying anemia and stroke syndromes are not completely understood [4], and it is believed that iron deficiency, may cause ischemic stroke by several potential mechanisms. Thrombocytosis is often associated with iron deficiency and microcytosis produces a reduction in the red cell deformability and could produce a hypercoagulable state [10]. The platelet count and function observed in iron-deficiency anemia could act synergistically to promote thrombus formation, especially in the setting of an underlying atherosclerotic disease [11]. Anemia may also worsen regional hypoxia in areas of decreased cerebral perfusion (so-called “anemic infarction”) [4].

The risk factors for carotid artery thrombus formation in the absence of atherosclerosis are not well characterized, although cases of carotid thrombus associated with iron-deficiency anemia have been reported previously [1]. Akins et al. reported 3 case reports in which young women with severe IDA and thrombocytosis secondary to menorrhagia developed carotid artery thrombosis [12]. Idbaih also stated that he followed 8 patients with spontaneous thrombosis of lesion-free carotid arteries and half of the patients with spontaneous thrombus had IDA, mostly secondary to menorrhagia [13]. Caplan et al. reported a young woman with severe iron- and folate-deficiency anemia with a non adherent carotid thrombus attached to a fatty streak [14]. Yarnell et al. described a patient with menorrhagia, hypochromic microcytic anemia, platelet count of 608,000 and a stroke, whose angiogram revealed a large intraluminal mass in the carotid bifurcation [3].

Thrombus formation requires platelet activation and aggregation onto an endothelial surface with subsequent fibrin deposition. A straightforward hypothesis is that the thrombocytosis leads to thrombus formation; however, the correlation between high platelet count and thrombosis is poor. Abnormal platelet activation and function are probably more important than absolute platelet count [12]. Thus iron deficiency may contribute to hypercoagulable state by affecting blood flow patterns within the vessels because of reduced deformability and increased viscosity of microcytic red blood cells [11]. Anemic patients need more blood flow to maintain compensation for the lack of oxygen. Therefore the increase in blood flow can cause endothelial damage, leading to platelet aggregation, causing a cascade for thrombus formation [1]. The presence of floating thrombus in a patient with clinical and MRI evidence of stroke represents a significant therapeutic dilemma and requires immediate decision about treatment. Thrombus in the internal carotid artery may resolve with medical management [2]. It is recommended to anticoagulate patients with or without antiplatelet drugs immediately. Initial anticoagulation for symptomatic intraluminal carotid artery thrombosis leads to a low rate of recurrent ischemic events, and that carotid revascularization if indicated can be safely performed in a delayed manner [15]. 

Assessment of iron profile should be performed when microcytic anemia is found. This is especially important because iron deficiency may occur as a result of an occult gastrointestinal bleeding following antiplatelet or anticoagulation therapy. Iron deficiency should be treated vigorously especially in patients with other significant thrombotic risk factors [2].
